# Gene coexpression network analysis and tissue-specific profiling of gene expression in jute (*Corchorus capsularis* L.)

**DOI:** 10.1186/s12864-020-06805-6

**Published:** 2020-06-16

**Authors:** Zemao Yang, Zhigang Dai, Xiaojun Chen, Dongwei Xie, Qing Tang, Chaohua Cheng, Ying Xu, Canhui Deng, Chan Liu, Jiquan Chen, Jianguang Su

**Affiliations:** Institute of Bast Fiber Crops, Chinese Academy of Agricultural Sciences / Key Laboratory of Stem-fiber Biomass and Engineering Microbiology, Ministry of Agriculture, Changsha, 410205 People’s Republic of China

**Keywords:** Comparative transcriptome analysis, Fiber crop, Jute, RNA-seq, WGCNA

## Abstract

**Background:**

Jute (*Corchorus* spp.), belonging to the Malvaceae family, is an important natural fiber crop, second only to cotton, and a multipurpose economic crop. *Corchorus capsularis* L. is one of the only two commercially cultivated species of jute. Gene expression is spatiotemporal and is influenced by many factors. Therefore, to understand the molecular mechanisms of tissue development, it is necessary to study tissue-specific gene expression and regulation. We used weighted gene coexpression network analysis, to predict the functional roles of gene coexpression modules and individual genes, including those underlying the development of different tissue types. Although several transcriptome studies have been conducted on *C. capsularis*, there have not yet been any systematic and comprehensive transcriptome analyses for this species.

**Results:**

There was significant variation in gene expression between plant tissues. Comparative transcriptome analysis and weighted gene coexpression network analysis were performed for different *C. capsularis* tissues at different developmental stages. We identified numerous tissue-specific differentially expressed genes for each tissue, and 12 coexpression modules, comprising 126 to 4203 genes, associated with the development of various tissues. There was high consistency between the genes in modules related to tissues, and the candidate upregulated genes for each tissue. Further, a gene network including 21 genes directly regulated by transcription factor OMO55970.1 was discovered. Some of the genes, such as *OMO55970.1*, *OMO51203.1*, *OMO50871.1*, and *OMO87663.1*, directly involved in the development of stem bast tissue.

**Conclusion:**

We identified genes that were differentially expressed between tissues of the same developmental stage. Some genes were consistently up- or downregulated, depending on the developmental stage of each tissue. Further, we identified numerous coexpression modules and genes associated with the development of various tissues. These findings elucidate the molecular mechanisms underlying the development of each tissue, and will promote multipurpose molecular breeding in jute and other fiber crops.

## Background

Jute (*Corchorus* spp.), belonging to the Malvaceae family, is an important natural fiber crop, second only to cotton [1]. Among more than 50 *Corchorus* species [2], only two (*C. capsularis* L. and *C. olitorius* L.) are grown commercially in subtropical and tropical regions [3]. Jute fibers have advantages such as good moisture absorption, fast water dispersion, corrosion resistance, and are mainly used in the textile industry to make clothes, decorations, packaging materials, and other products [3, 4]. Jute is a multipurpose economic crop, and each tissue has its particular usage. For example, jute stalks can be used to make paper and to provide fuel, activated carbon, environmental protection materials, and building composite materials [3]. The leaves can be used as green vegetables and animal feed, and to produce skin care products and herbal medicine [5]. The seeds can be used to extract industrial oil and other products [6]. The versatility of jute in the marketplace will continue to expand, especially in environmental protection, vegetable production, and facial mask manufacturing [7]. These many benefits derive from the different chemical, physical, and biological properties of its various tissues, which are under tissue-specific gene expression control.

Understanding the expression and regulation of genes in different tissues will help us to elucidate the molecular mechanisms underlying the development of these tissues [8]. With the rapid development of high-throughput sequencing technology and bioinformatics, tissue-specific gene expression and regulation analyses have been carried out on many crops [9–11]. In particular, weighted gene coexpression network analysis (WGCNA) has recently been widely used to predict the functional roles of gene coexpression modules and individual genes underlying the differences between tissues [12–14]. For example, WGCNA or comparative transcriptomic analysis revealed coexpression modules and dynamics in gene expression involved in stress response [12], seed development [13], and floral bud development [14] in various tissues of crop plants, etc.. WGCNA has become a fascinating integrated and systematic genome-wide approach, focusing on elucidating biological networks and gene function [15]. Recently, high-throughput sequencing technology has greatly promoted the study of jute molecular biology and genetics. For *C. capsularis*, many molecular markers including single nucleotide polymorphisms and simple sequence repeats have been developed through high-throughput sequencing [4, 16–18]. Several transcriptome studies have been reported in *C. capsularis*. These uncovered numerous differentially expressed unigenes involved in vegetative growth and development [19], abiotic stress [20] and bast fiber development [21]. However, a systematic and comprehensive transcriptome analysis has not yet been reported for *C. capsularis*.

In this study, we performed a transcriptome analysis of different *C. capsularis* tissues in two different developmental stages. Our objective was to understand the molecular mechanisms underpinning the development of different tissue types, and to promote multipurpose molecular breeding in jute and other fiber crops.

## Results

### Transcriptome sequencing

We sequenced 19 RNA samples from jute (Yueyuan5hao) stem bast, leaf, fruit, and flower tissues during differential developmental stages. A total of 943.45 million high-quality reads were generated. Because jute flowers are very small, many flowers were required in order to obtain enough RNA for sequencing studies. Therefore, we combined many flowers for sequencing, whereas the other tissues were sequenced using three biological replicates. The smallest amount of sequencing data was obtained for flower tissue (54.22 million clean reads). We obtained clean reads for all other tissues, ranging from 145.26 to 152.42 million reads. We mapped the clean reads to the *C. capsularis* reference genome (CCACVL1_1.0); most clean reads from each tissue (> 92.45%) were aligned uniquely to the reference genome (Table 1).

**Table 1:** RNA sequencing statistics for tissues during two developmental stages in jute

Sample name	Clean reads (Millions)	Clean bases(Gb)	Q20(%)	Uniquely mapped (Millions)	Uniquely mapped rate (%)
BVGP1	51.27	7.70	97.15	47.93	93.48
BVGP2	47.76	7.16	97.28	44.75	93.70
BVGP3	51.17	7.68	96.81	47.94	93.70
Total of BVGP	150.20	22.54	97.08	140.63	93.62
BFP1	50.29	7.54	97.10	47.40	94.27
BFP2	45.46	6.82	97.02	42.18	92.79
BFP3	53.74	8.06	97.35	50.66	94.27
Total of BFP	149.48	22.42	97.15	140.24	93.82
LVGP1	45.88	6.88	96.99	42.82	93.33
LVGP2	46.00	6.90	96.68	42.40	92.17
LVGP3	53.38	8.00	96.66	49.08	91.94
Total of LVGP	145.26	21.78	96.78	134.29	92.45
LFP1	52.76	7.92	97.13	48.91	92.69
LFP2	48.52	7.28	97.11	45.19	93.14
LFP3	51.15	7.68	97.10	47.26	92.40
Total of LFP	152.43	22.88	97.11	141.36	92.74
FT1_1	52.53	7.88	97.13	49.64	94.48
FT1_2	47.48	7.12	96.88	43.99	92.66
FT1_3	45.50	6.82	96.87	42.24	92.83
Total of FT1	145.51	21.82	96.96	135.87	93.37
FT2_1	52.58	7.88	96.95	49.10	93.38
FT2_2	46.09	6.92	97.60	42.96	93.21
FT2_3	47.69	7.16	97.50	44.21	92.70
Total of FT2	146.36	21.96	97.35	136.27	93.10
MF	54.22	8.14	97.29	50.98	94.03
Total	943.45	141.54		879.63	

*MF* mature flowers, *LVGP* leaf tissues of vegetative growth period, *LFP* leaf tissues of flowering period, *FT1* Fruits < 0.8 cm in diameter, *FT2* Fruits > 0.8 cm in diameter, *BVGP* bast of vegetative growth period, *BFP* bast of flowering period. Each tissue with three biological replicates

### Global transcriptome analysis of jute

**Fig. 1:**
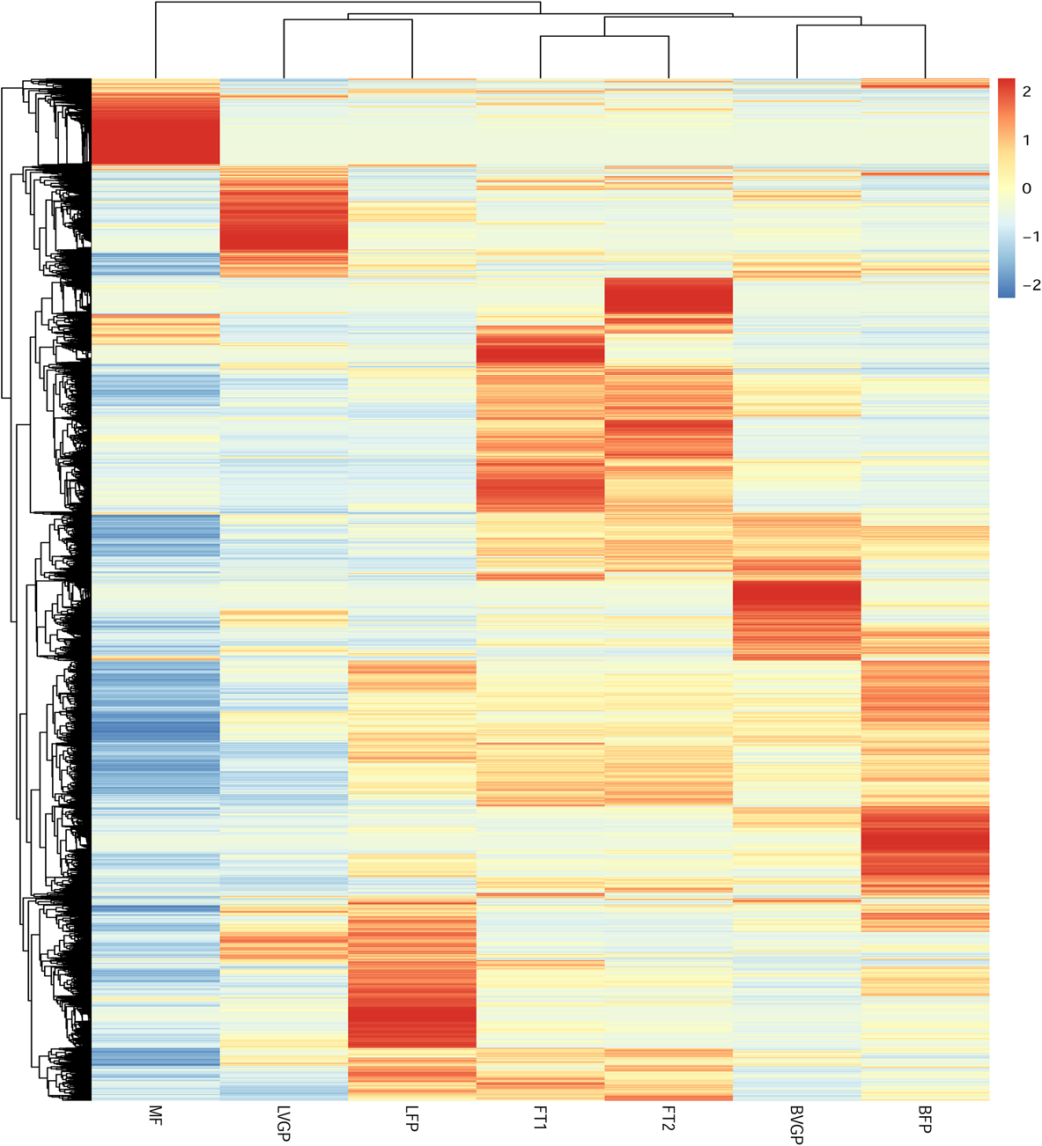
Hierarchical cluster analysis of the jute genes that we analyzed. The analysis is based on reads per kilobase of transcript per million mapped reads (RPKM). MF, mature flowers; LVGP, leaf tissues of vegetative growth period; LFP, leaf tissues of flowering period; FT1, Fruits < 0.8 cm in diameter; FT2, Fruits > 0.8 cm in diameter; BVGP, bast of vegetative growth period; BFP, bast of flowering period

To assess the number of genes expressed in the various tissue types at different stages, we analyzed the reads per kilobase of exon model per million reads (RPKM) of all 29,605 genes identified in this study. An RPKM value greater than one was set as the criteria for gene expression. In stem bast tissues, there was transcriptional activity for 18,320 and 18,268 genes, during the vegetative growth period (“bast tissue during the vegetative growth period”, BVGP) and flowering period (“bast tissue during the flowering period”, BFP), respectively (Additional files 1 and 2: Tables S1–2); in leaf tissues, 17,480 and 18,126 genes were expressed in these two stages, respectively (Additional files 3 and 4: Tables S3–4). During the flowering period, fruits were categorized into two developmental stages (diameter < 0.8 cm, hereafter “FF1”; or diameter > 0.8 cm, hereafter “FF2”), and were used for RNA sequencing; 19,396 and 19,509 genes, respectively, were expressed in these developmental stages (Additional files 5 and 6: Tables S5–6). Furthermore, 17,842 expressed genes (Additional file 7: Table S7) were identified in flowers. In total, 14,943 genes (Additional file 8: Table S8) were expressed across all tissues during the vegetative growth period and flowering period. Differences in gene expression between tissues were visualized using hierarchical cluster analysis based on the RPKM values of the 29,605 genes (Fig. 1).

### Comparative transcriptome analysis of the different tissues and developmental stages

**Fig. 2:**
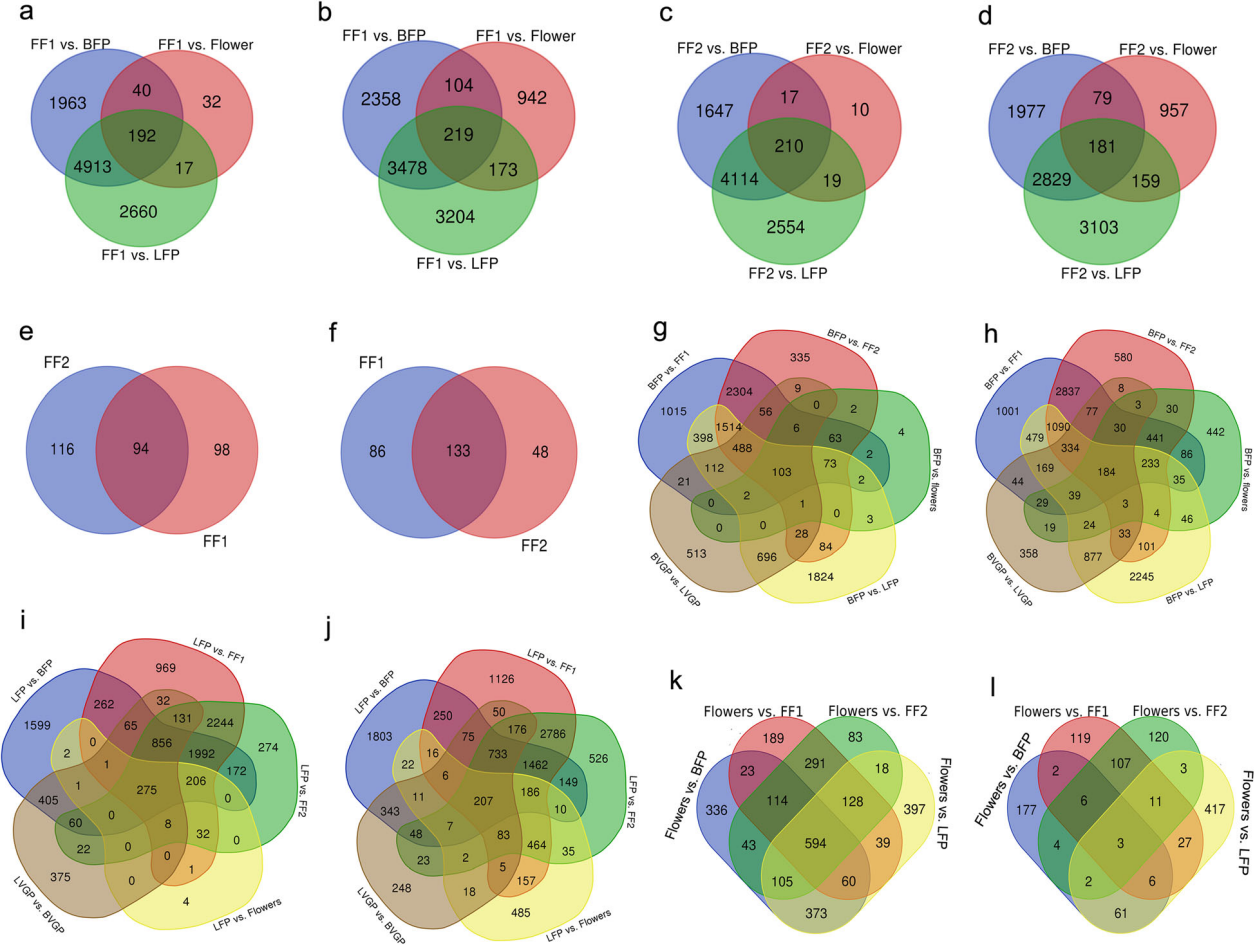
Venn diagram of differential gene expression between various jute tissues. **a**-**d** Upregulated (**a** and **c**) and downregulated (**b** and **d**) genes in fruit tissues (FF1 and FF2) compared with other tissues at each developmental stage. **e**, **f** Genes identified as upregulated (**e**) and downregulated (**e**) in fruit tissue (FF1 and FF2) compared with all other tissues at each developmental stage. **g**, **h** Upregulated (**g**) and downregulated (**h**) genes in stem bast tissues compared with other tissues of the same developmental stage. **i**, **j** Upregulated (**i**) and downregulated (**j**) genes in leaf tissues compared with other tissues at the same developmental stage. **k**, **l** Upregulated and downregulated genes in flower tissues compared with other tissues at the same stage. MF, mature flowers; LVGP, leaf tissues of vegetative growth period; LFP, leaf tissues of flowering period; FT1, fruits < 0.8 cm in diameter; FT2, fruits > 0.8 cm in diameter; BVGP, bast of vegetative growth period; BFP, bast of flowering period

We identified the candidate differentially expressed genes (DEGs) for each tissue by comparison with other tissues at the same developmental stage. Relative to leaf tissues (“leaf tissues during the vegetative growth period”, LVGP), we identified 2035 upregulated and 2231 downregulated genes in BVGP (Additional files 9 and 10: Tables S9–10). In FF1, there were 7108 upregulated and 6059 downregulated genes, relative to BFP; 7782 upregulated and 7074 downregulated genes, relative to leaf tissues during the flowering period (LFP); 281 upregulated and 1438 downregulated genes, relative to flowers; and 192 upregulated and 219 downregulated genes, relative to all other tissue types (Fig. 2a and b). In FF2, there were 5988 upregulated and 5067 downregulated genes, relative to BFP; 6897 upregulated and 6272 downregulated genes, relative to LFP; 256 upregulated and 1376 downregulated genes, relative to flowers; and 210 upregulated and 181 downregulated genes, relative to all other tissue types (Fig. 2c, d). In total, 94 upregulated and 133 downregulated genes were identified in fruit during the FF1 and FF2 developmental stages (Fig. 2e and f). In BFP, we identified 6059 upregulated and 7108 downregulated genes, relative to FF1; 5067 upregulated and 5988 downregulated genes, relative to FF2; 5328 upregulated and 5896 downregulated genes, relative to LFP; and 261 upregulated and 1648 downregulated genes, relative to flowers. In total, 103 upregulated and 184 downregulated genes were identified in stem bastduring the vegetative growth period and flowering period (Fig. 2g and h). In total, 275 upregulated and 207 downregulated genes were identified by comparing leaf tissues with other organ tissues during the vegetative growth period and flowering period (Fig. 2i and j). The fewest DEGs (< 3000 in total) were found in flower tissues relative to other tissues (Fig. 2k and l).

### Identification of gene coexpression modules

**Fig. 3:**
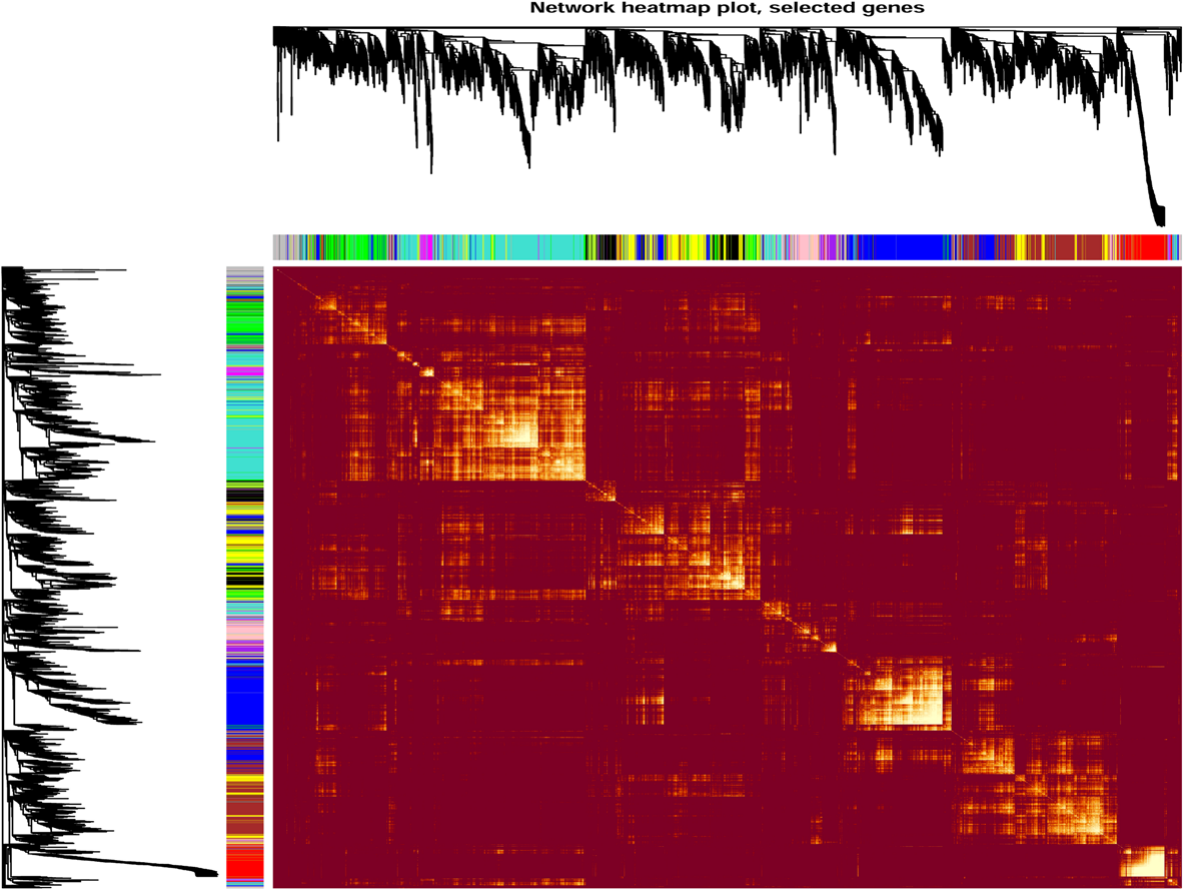
The twelve coexpression modules, comprising 126 to 4203 jute genes. The modules were identified using weighted gene coexpression network analysis (WGCNA). The heatmap depicted adjacencies or topological overlaps, with light colors denoting higher adjacency (correlation), with red colors denoting low adjacency (correlation). The gene dendrograms and module colors are plotted along the top and left side of the heatmap. Each color represents a module

**Fig. 4:**
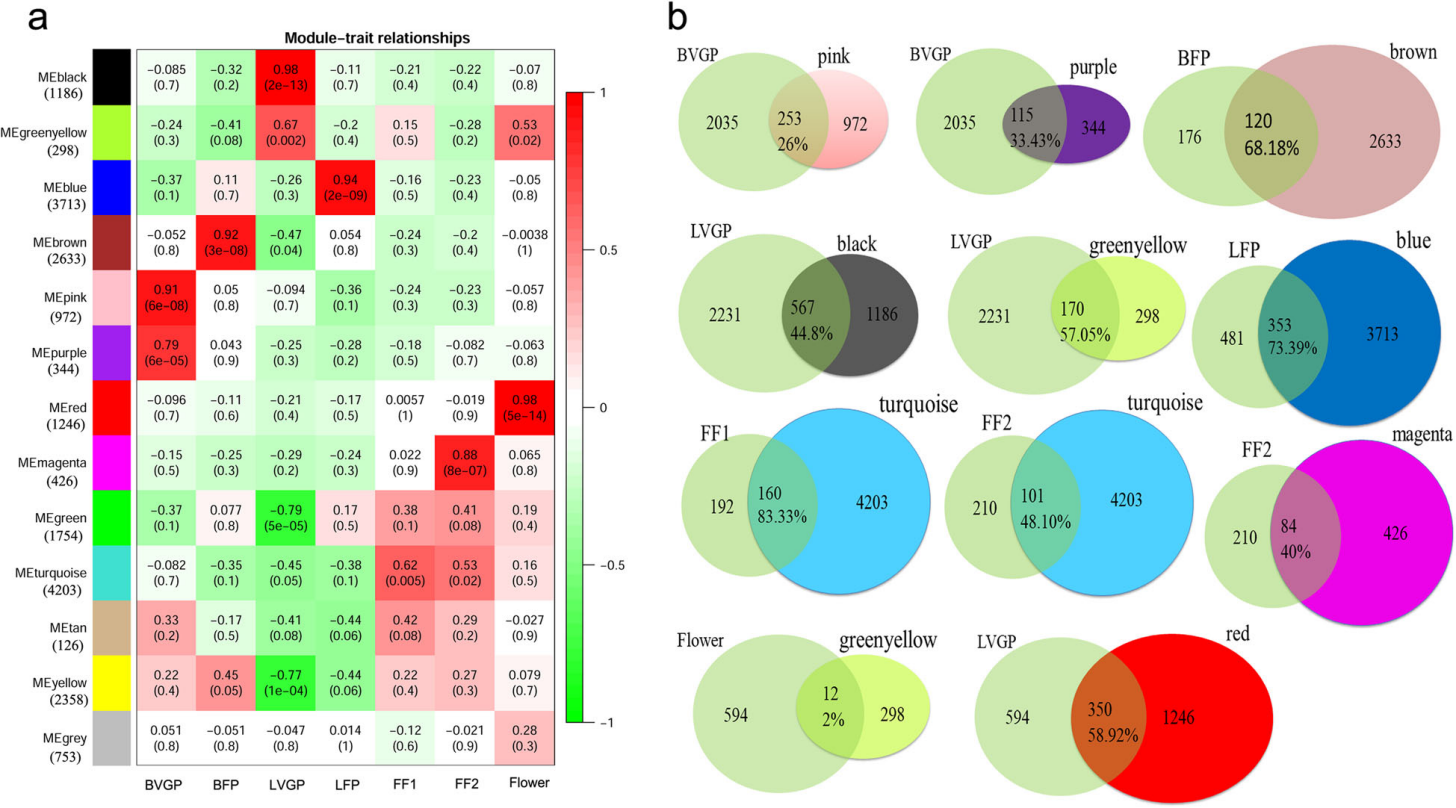
Coexpression module and gene comparison analyses. We compared the genes in modules related to traits to the candidate upregulated genes. **a** Correlations between the modules and tissues at two different developmental stages. **b** Overlap between genes in modules related to traits and candidate upregulated genes. MF, mature flowers; LVGP, leaf tissues of vegetative growth period; LFP, leaf tissues of flowering period; FT1, fruits < 0.8 cm in diameter; FT2, fruits > 0.8 cm in diameter; BVGP, bast of vegetative growth period; BFP, bast of flowering period

To identify genes and coexpression modules with similar expression profiles related to the development of different tissues, we carried out a WGCNA. To avoid spurious results, low-expression genes (average RPKM< 1) were excluded. In total, 20,012 genes were used in this analysis, and 12 coexpression modules comprising 126 to 4203 genes were identified; and there is a higher correlation among genes in modules (Fig. 3). Further, we investigated the associations between each module and each tissue at different developmental stages, using correlation analysis. Only one module was related to LFP (related module: blue), FF1 (related module: turquoise), and BFP (related module: brown); two modules were related to BVGP (related module: pink and purple), FF2 (related module: turquoise and magenta), LVGP (related module: black and greenyellow), and flowers (related module: greenyellow and red) (Fig. 4a). The turquoise module correlated with both FF1 and FF2. By comparing the genes in the modules related to particular traits to the candidate upregulated genes for each tissue type (defined as comparison group), we found that the candidate upregulated genes and the genes in each module were highly consistent for each comparison group. The ratio of overlapping genes between each comparison group was greater than 20% for almost all combinations, except the combination of flowers and the greenyellow module (2%) (Fig. 4b).

We performed Kyoto Encyclopedia of Genes and Genomes (KEGG) analysis for the overlapping genes for each comparison group. The terms ‘protein processing in endoplasmic reticulum’, ‘sesquiterpenoid and triterpenoid biosynthesis’, ‘plant hormone signal transduction’, and ‘glycolysis/gluconeogenesis’, were enriched in stem bast tissues. In addition to other terms, the terms ‘phenylpropanoid biosynthesis’, ‘biosynthesis of secondary metabolites’, and ‘flavonoid biosynthesis’ were enriched in the fruit; ‘pentose and glucuronate interconversions’ and ‘phenylalanine metabolism’ were enriched in the flowers; and ‘metabolic pathways’ and ‘photosynthesis’ were enriched in the leaf tissues (Additional files 11 and 12: Fig. S1–2).

### Identification of genes in coexpression modules associated with fiber tissues

**Fig. 5:**
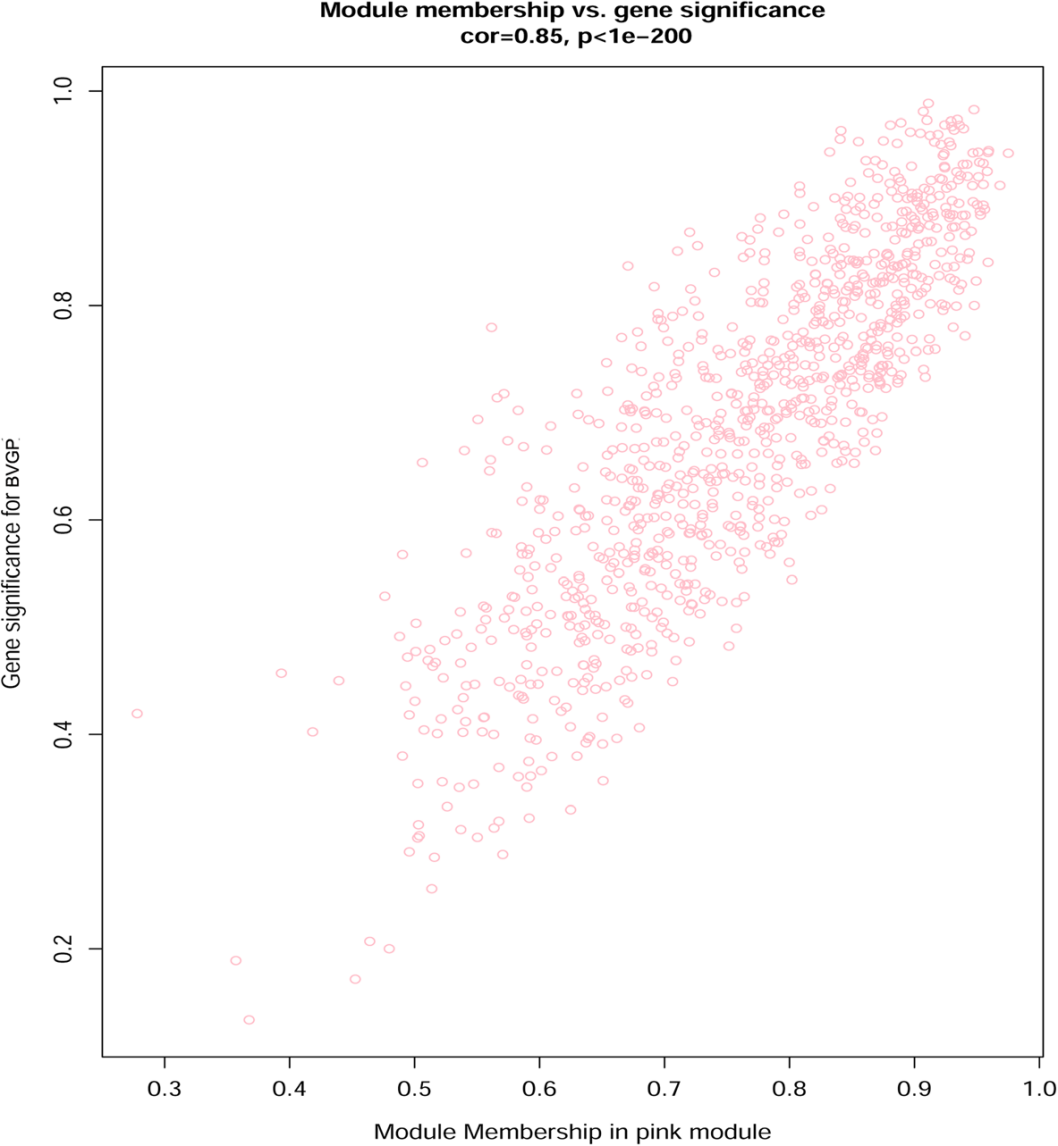
Scatterplot of Gene Significance (GS) score versus module membership (MM) in the pink module. This is for stem bast tissue during the vegetative growth period

**Fig. 6:**
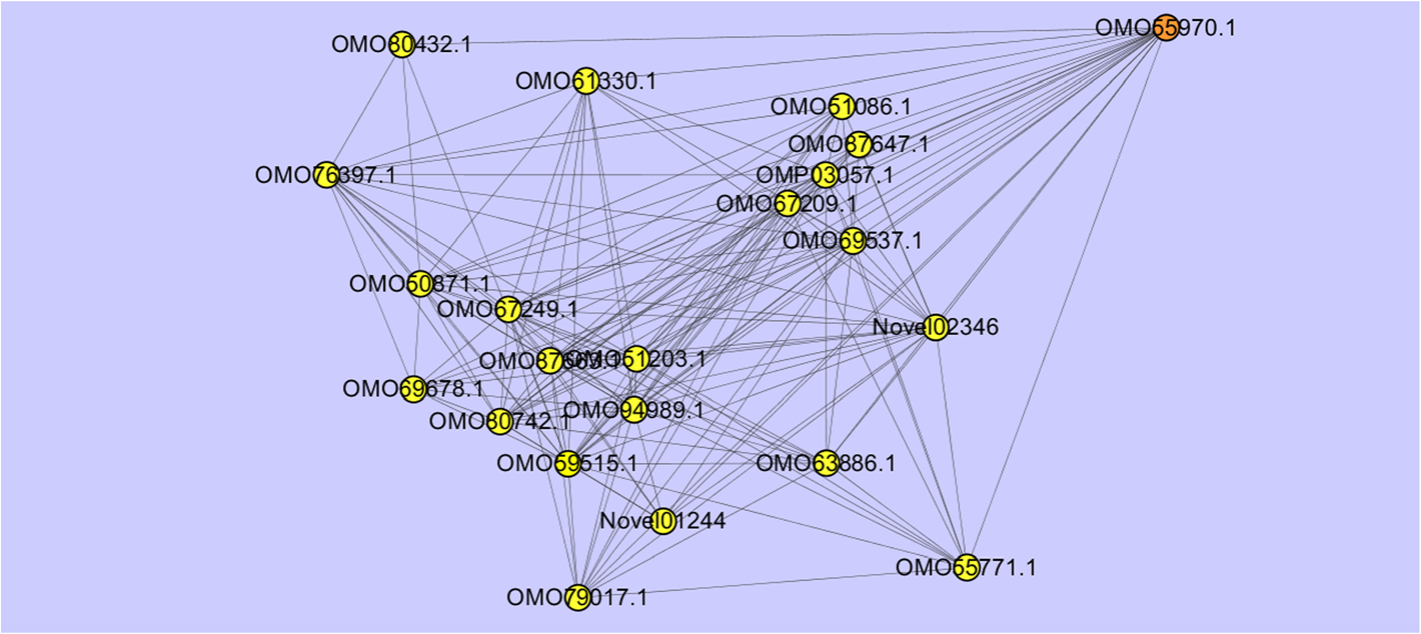
A network of 21 genes directly linked to transcription factor OMO55970.1. This transcription factor is associated with genes in the pink module that code for fiber tissues

The vegetative growth period is the period of jute fiber development and rapid thickening of stem bast. Based on the WGCNA results, the pink module related with BVGP. We evaluated the correlation between the expression of genes and stem bast tissues, and define this value as the Gene Significance (GS) score. We also assessed the correlation of the pink module with the gene expression profiles, based on this correlation, we defined module membership (MM) in the pink module. GS and MM were closely correlated (cor = 0.85, *p* < 1e^− 200^) in the pink module for stem bast tissue (Fig. 5), reflecting the strong correlation between stem bast tissue and the pink module genes. We identified 253 upregulated genes in stem bast that also occurred within the pink module, during the vegetative growth period. We further analyzed and constructed a coexpression network for these genes. We focused on a transcription factor gene (*OMO55970.1*), which was directly linked to 21 other genes (Fig. 6). Fourteen of these genes were included among the 253 common genes (Table 2). Some of these 14 genes were involved in the development of stem bast and fiber, with very high GS.BVGP, and MM.pink values. For example, *OMO50871.1* is an epidermal patterning factor, OMO51203.1 is related to glucose metabolism, and *OMO87663.1* is a wall-associated receptor kinase.

**Table 2:** Gene significance and membership of the pink module, for 15 jute genes including *OMO55970.1* and genes directly linked to it

Gene name	GS.BVGP	P.GS.BVGP	MM.pink	P.MM.pink	log_2_FC	P-adj	Annotation
*OMO55970.1*	0.47	0.0448	0.59	0.0079	1.84	0.0052	sp|Q39237|TGA1_ARATH Transcription factor TGA1
*Novel02346*	0.64	0.0034	0.68	0.0014	2.52	0.0005	sp|P04793|HSP13_SOYBN 17.5 kDa class I heat shock protein
*OMO50871.1*	0.78	0.0001	0.87	0.0000	1.86	0.0332	sp|Q9T068|EPFL2_ARATH EPIDERMAL PATTERNING FACTOR-like protein
*OMO51203.1*	0.84	0.0000	0.89	0.0000	1.82	0.0056	sp|Q9LTA3|U91C1_ARATH UDP-glycosyltransferase 91C1
*OMO55771.1*	0.59	0.0081	0.60	0.0067	2.76	0.0000	sp|Q94CH6|EXL3_ARATH GDSL esterase/lipase EXL3
*OMO59515.1*	0.77	0.0001	0.80	0.0000	3.24	0.0000	sp|Q84TH5|AB25G_ARATH ABC transporter G family member 25
*OMO63886.1*	0.56	0.0118	0.70	0.0010	5.24	0.0497	
*OMO67249.1*	0.77	0.0001	0.83	0.0000	1.60	0.0162	
*OMO69537.1*	0.70	0.0010	0.70	0.0008	1.67	0.0271	sp|Q01JD1|SPL7_ORYSI Squamosa promoter-binding-like protein 7
*OMO76397.1*	0.60	0.0072	0.73	0.0004	2.14	0.0008	sp|Q9FKW0|RNG1A_ARATH Putative E3 ubiquitin-protein ligase RING1a
*OMO79017.1*	0.57	0.0104	0.59	0.0076	4.70	0.0317	
*OMO80742.1*	0.87	0.0000	0.90	0.0000	3.33	0.0001	
*OMO87663.1*	0.87	0.0000	0.94	0.0000	2.10	0.0010	sp|Q8RY67|WAKLO_ARATH Wall-associated receptor kinase-like 14
*OMO94989.1*	0.85	0.0000	0.83	0.0000	2.06	0.0038	sp|O04212|Y2090_ARATH Putative ABC1 protein
*OMP03057.1*	0.63	0.0038	0.68	0.0014	3.67	0.0000	

These genes were differentially expressed between the jute bast and other tissues

### Validation of the differential gene expression results

To validate the RNA-seq results, qRT-PCR analysis was performed for 12 genes during the vegetative growth period. The genes showed differential expression when comparing stem bast with leaf tissue, consistent with the results obtained by the RNA-seq analysis (Additional file 13: Fig. S3). In addition, we compared DEGs identified in bast tissue during the vegetative growth period in our study with the DEGs identified in fibre cells which were included in bast tissue by comparing fibre cells with seedling reported by Islam et al. A total of 714 upregulated and 837 downregulated genes were discovered in the both studies (Additional files 14 and 15: Tables S11–12), accounting for approximately 35% (714/2035) and 38% (837/2231) of upregulated and downregulated genes identified in bast tissue during the vegetative growth period in our study.

## Discussion

Knowing how genes are expressed and regulated in various tissues is the basis of studying gene function, and is required for molecular breeding. The study of gene expression profiles of various tissues during different developmental stages has been reported for many crops [22, 23]. However, a systematic and comprehensive transcriptome has not yet been reported for *C. capsularis*, and transcription information for various tissues is lacking. In the study, we compared the transcriptomes of four tissue types during two growth stages, revealing many genes and marked distinctions in gene expression. Tissues were clustered together according to their gene expression signatures, consistent with previous reports [23, 24].

Comparative transcriptome analysis is a useful and conventional method for investigating spatiotemporal pattern of gene expression [23, 25–27]. However, it is difficult to examine the relationships between DEGs and tissues using differential gene expression analysis. Therefore, the systematic analytical methods are called for comprehensively extracting information from transcriptome data. WGCNA is a popular and useful approach for revealing meaningful relationships between gene modules and biological processes, and between genes and traits [13, 14]. In this study, we first identified the genes that were differentially expressed in each tissue compared with other tissues in the same developmental period, then identified the genes that were consistently up- or down regulated in different periods for each tissue. These findings will help us to understand the development of different tissues. In particular, genes that are consistently up- and downregulated might play crucial roles in the metabolism, growth, and development of each tissue; these should be the focus of future studies.

We then identified 12 coexpression modules comprising 126 to 4203 genes, that were associated with the development of various tissues, using WGCNA. We compared the genes in modules related to tissues, to the candidate upregulated genes in each tissue, and found that they were highly consistent (Fig. 4b). This corroborated our analysis of the correlation between the coexpression modules and the gene expression profiles of each tissue. Furthermore, the KEGG analysis for the overlapping genes for each comparison group showed that the various tissue types have different enrichment pathways. This provides evidence that the genes that code for the pathways have variable spatiotemporal pattern of expression in the tissues. Some similar results have been found in previous studies on jute pathway enrichment, for example, ‘glycolysis/gluconeogenesis’ was enriched in stem bast tissue, which associated with bast fibre synthesis [19, 28];

We verified that the WGCNA was suitable for the analysis of gene expression modules in the various tissues that we studied. Additionally, 253 genes that were upregulated in stem bast also occurred within the pink module, during the vegetative growth period. Among these genes, transcription factor OMO55970.1 was directly linked to 21 other genes, some of them showing very high GS.BVGP and MM.pink correlation and directly involved in the development of stem bast and fiber according to gene annotation [28]. In particular, OMO55970.1, a TGA1 transcription factor, is a candidate transcription factor for fiber differentiation [29]. OMO50871.1 is an epidermal patterning factor; OMO51203.1 is related to glucose metabolism; and OMO87663.1 is a wall-associated receptor kinase which play a crucial role in correct cell-wall synthesis [30]. Our results suggest that these genes are of great significance for the formation of stem bast and fiber, and that they should be the focus of future research.

## Conclusion

This is the first systematic and comprehensive transcriptome analysis for *Corchorus capsularis*. To describe the transcriptomes associated with various tissue types and developmental stages, we performed a comparative transcriptome and coexpression modules analysis. We compared the genes in modules related to tissues to the candidate upregulated genes for each tissue, and found that they were highly consistent. These results constituted a systematic and complete database of gene expression of jute various tissue types and tissue development. We identified a gene network of 21 genes that was directly regulated by transcription factor OMO55970.1. Some of these genes, such as *OMO55970.1, OMO51203.1, OMO50871.1*, and *OMO87663.1*, were immediately involved in the development of stem bast and fiber. These genes should be the focus of future research. In summary, these findings help to elucidate the molecular mechanisms of tissue development in jute, and to promote multipurpose molecular breeding in jute and other fiber crops.

## Methods

### Plant growth and transcriptome sequencing

The *C. capsularis* used in this study was a variety (Yueyuan5hao). It originally came from Guangdong Province, China, and stored in the National Bast Fiber Germplasm Middle-term Storage of China (our lab, in Changsha, Hunan province, China). In total, 20 plants were planted in a well-ventilated greenhouse, in 10 pots with two plants per pot. During the entire growth period, the environmental conditions of the soil and fertilizer in each pot were kept consistent, to ensure minimal differences in plant growth. When the plants had grown to approximately 1 m (vegetative growth period), stem bast and leaf tissues were collected for RNA extraction. During the flowering and fruiting period, tissues including leaves, stem bast, fruits with diameter < 0.8 cm, fruits with diameter > 0.8 cm, and flowers were collected for RNA extraction. Three replicates of each tissue were collected, except of flowers. Owing to the small size of the flowers, we sampled enough amount for mixed sequencing. Total RNA was extracted from 19 tissue samples using Trizol (Invitrogen, Santa Clara, CA, USA) following the manufacturer’s protocol. RNA quality assessment and construction of sequencing libraries were performed according to Yang et al. [31]. In total, 19 sequencing libraries were generated, using NEBNext Ultra RNA Library Prep Kit for Illumina (NEB, USA). RNA sequencing was carried out on an Illumina HiSeq 2500 System.

### Read mapping and differential gene expression analysis

Firstly, raw reads were processed in Perl using in-house scripts, to obtain clean, high quality reads by removing adapter sequences and low-quality reads. All downstream analyses were performed using the clean reads. We downloaded the reference genome of *C. capsularis* (CCACVL1_1.0) from the NCBI database [28]. We then used Bowtie v2.0.6 [32] to build the reference genome index, TopHat v2.0.9 [33] to map clean reads to the reference genome, and HTSeq v0.6.1 to count the number of reads aligned to each gene [34]. To calculate gene expression levels, we assessed the RPKM of each gene based on the number of reads mapped to the gene and the length of the gene. Finally, differential gene expression analysis was performed using a model based on the negative binomial distribution, using the R package DESeq (1.10.1) [35]. Genes with an adjusted *P*-value < 0.05 were considered to be differentially expressed. *P*-values were adjusted to reduce the false discovery rate using the Benjamini-Hochberg procedure [35].

### Identification of gene coexpression modules

To identify gene clusters associated with various tissue types, we performed a WGCNA using the R package WGCNA [36], using RPKM values from the 19 tissue samples. First, we clustered all samples to exclude any obvious outliers (cutHeight = 3000). We then performed network construction and module detection using an automatic 1-step network construction and module detection function. We constructed a weighted gene network using thresholding power β (β = 1 to 30) to calculate adjacency among the genes. Further, we chose the soft thresholding power (β = 16) to construct a network based on the criterion of approximate scale-free topology, using mergeCutHeight = 0.25 and minModuleSize = 30. We estimated the association between genes and various tissues using the Gene Significance (GS) score (whereby each tissue is considered a quality trait), the correlation of the module and the gene expression profile (MM) and the correlation of module with traits (module eigengene). This enabled us to identify the expression modules and genes that are closely related to each tissue. Lastly, the WGCNA results were exported to Cytoscape software to construct a gene coexpression network [37].

### Pathway enrichment analysis of differentially expressed genes

To examine the metabolic pathways associated with the development of different tissues, we first identified the genes that were both included in modules related to specific traits, and were upregulated in each tissue relative to the other tissues at the same stage. Second, we performed a KEGG enrichment analysis, using KOBAS software, for the genes that were identified both in each tissue and each module [38, 39]. Third, we selected the 20 most significantly enriched pathways to visualize in the maps. When fewer than 20 pathways were significantly enriched, all were displayed.

### Validation of differential gene expression

To validate the RNA-seq results, we compared a set of genes expression in this study with the report by Islam et al. [28], and analyzed the expression of 12 genes (7 that were directly regulated by transcription factor OMO55970.1, and five randomly selected from among the DEGs expressed in leaves and stem bast tissues during the vegetative growth period) by qRT-PCR, using three independent biological and three technological replicates. We used the jute elongation factor-alpha (*ELF*) gene as an endogenous control. The primer sequences of the DEGs and *ELF* are listed in Additional file 16: Table S13. The qRT-PCR was performed according to Yang et al. [20].

## Supplementary information

**Additional file 1: Table S1.** Transcriptional genes that were identified in stem bast tissue during the vegetative growth period (BVGP).

**Additional file 2: Table S2.** Transcriptional genes that were identified in bast tissue during the flowering period (BFP).

**Additional file 3: Table S3.** Transcriptional genes that were identified in leaf tissues during the vegetative growth period (LVGP).

**Additional file 4: Table S4.** Transcriptional genes that were identified in leaf tissues during the flowering period (LFP).

**Additional file 5: Table S5.** Transcriptional genes that were identified in fruit < 0.8 cm in diameter (FF1).

**Additional file 6: Table S6.** Transcriptional genes that were identified in fruit > 0.8 cm in diameter (FF2).

**Additional file 7: Table S7.** Transcriptional genes that were identified in flower tissues.

**Additional file 8: Table S8.** Transcriptional genes that were identified in all tissues during differential stages.

**Additional file 9: Table S9.** Upregulated genes that were identified by comparing bast tissue during the vegetative growth period (BVGP) with leaf tissue during the vegetative growth period (LVGP).

**Additional file 10: Table S10.** Downregulated genes that were identified by comparing bast tissue during the vegetative growth period (BVGP) with leaf tissue during the vegetative growth period (LVGP).

**Additional file 11: Figure S1.** Enriched terms for the genes that overlapped between the gene modules related to traits and the candidate upregulated genes, obtained using KEGG enrichment analysis.

**Additional file 12: Figure S2.** Enriched terms for the genes that overlapped between the gene modules related to traits and the candidate upregulated genes, obtained using KEGG enrichment analysis.

**Additional file 13: Figure S3.** RNA-seq and qRT-PCR analysis results for the genes directly regulated by transcription factor (OMO55970.1), and for the five genes that were randomly selected from among the genes that were differentially expressed between the bast tissueand the leaf tissue during the vegetative growth period.

**Additional file 14: Table S11.** Common upregulated genes identified in bast tissue during the vegetative growth period in our study and in fibre cells which were included bast tissue identified by comparing fibre cells with seedling reported by Islam et al. [28].

**Additional file 15: Table S12.** Common downregulated genes identified in bast tissue during the vegetative growth period in our study and in fibre cells which were included bast tissue identified by comparing fibre cells with seedling reported by Islam et al. [28].

**Additional file 16: Table S13.** The primer sequences used for ELF, for the seven genes that were directly regulated by transcription factor (OMO55970.1), and for the five genes that were randomly selected from among the differentially expressed genes.

## Data Availability

All data generated or analyzed during this study are included in this published article or its supplementary information files. Sequencing raw data have been submitted to NCBI under accession number PRJNA612788.

## Abbreviations

WGCNA: Weighted gene coexpression network analysis
RPKM: Reads per kilobase of exon model per million reads
KEGG: Kyoto Encyclopedia of Genes and Genomes
GS: Gene Significance
MM: Module membership
qRT-PCR: quantitative reverse transcriptase polymerase chain reaction
RNA-seq: RNA sequencing
DEGs: Differentially expressed genes
*ELF*: Elongation factor-alpha
MF: Mature flowers
LVGP: Leaf tissues of vegetative growth period
LFP: Leaf tissues of flowering period
FT1: Fruits < 0.8 cm in diameter
FT2: Fruits > 0.8 cm in diameter
BVGP: Bast of vegetative growth period
BFP: Bast of flowering period
