# Supplementary figures and images for: Gene coexpression network analysis and tissue-specific profiling of gene expression in jute (Corchorus capsularis L.)
Source: BMC Genomics. 2020 Jun 16;21:406. doi: 10.1186/s12864-020-06805-6 (PMC7298812; doi:10.1186/s12864-020-06805-6)

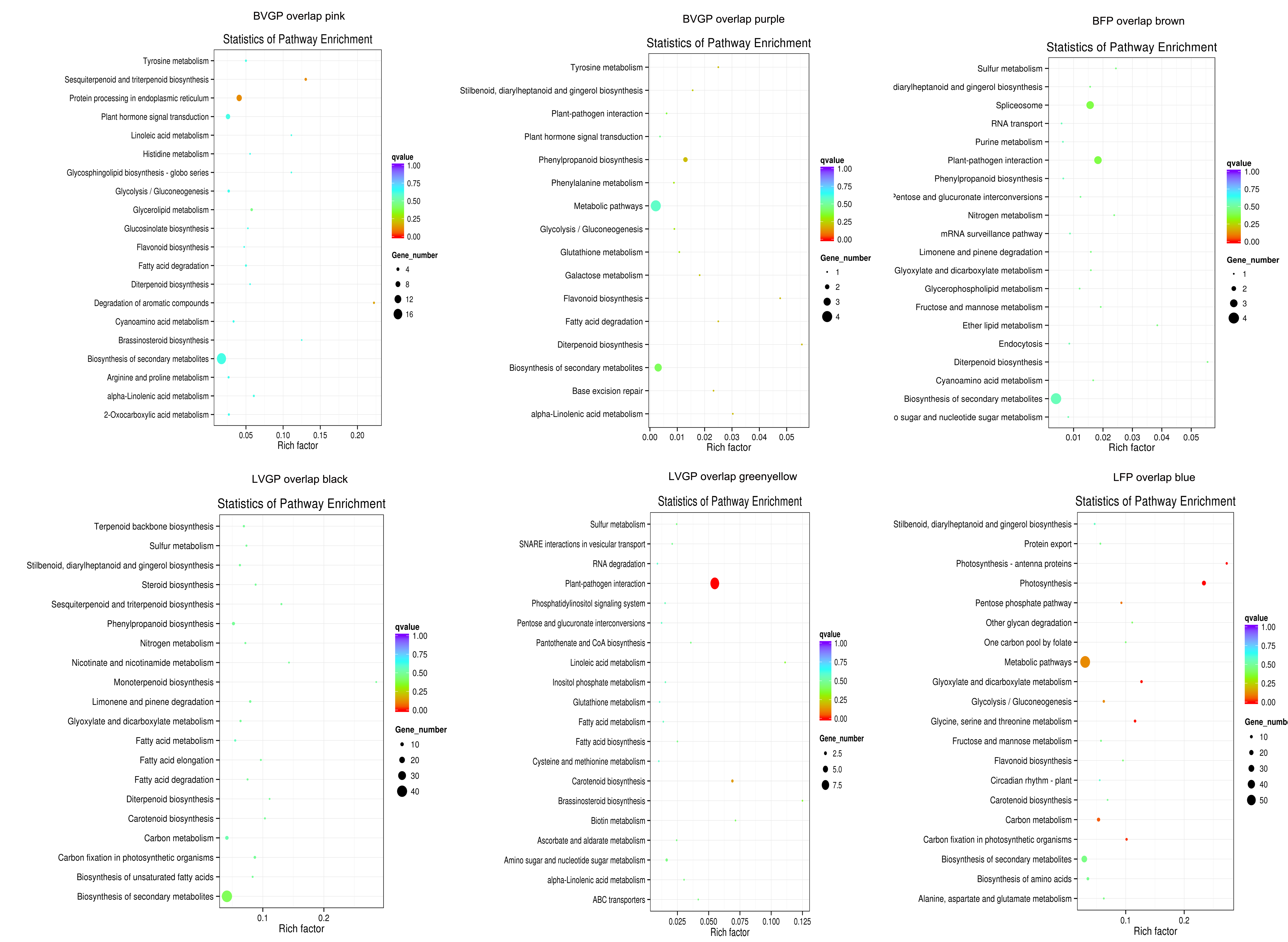

Supplement: Supplementary file 11 — Additional file 11: Figure S1. Enriched terms for the genes that overlapped between the gene modules related to traits and the candidate upregulated genes, obtained using KEGG enrichment analysis. [file 12864_2020_6805_MOESM11_ESM.tif]

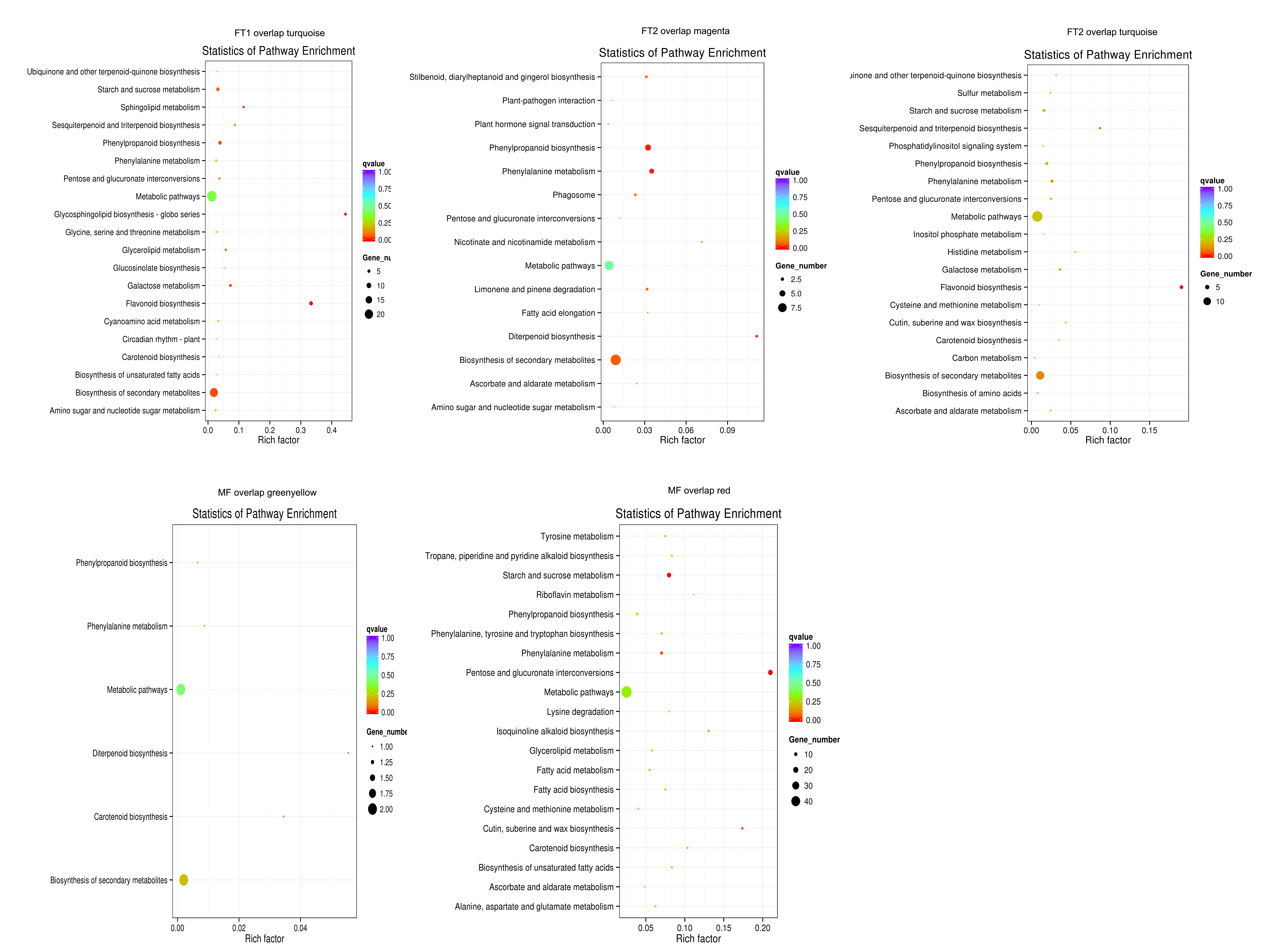

Supplement: Supplementary file 12 — Additional file 12: Figure S2. Enriched terms for the genes that overlapped between the gene modules related to traits and the candidate upregulated genes, obtained using KEGG enrichment analysis. [file 12864_2020_6805_MOESM12_ESM.tif]
